# Repeatability of shear wave elastography in liver fibrosis phantoms—Evaluation of five different systems

**DOI:** 10.1371/journal.pone.0189671

**Published:** 2018-01-02

**Authors:** Anesa Mulabecirovic, Anders Batman Mjelle, Odd Helge Gilja, Mette Vesterhus, Roald Flesland Havre

**Affiliations:** 1 Department of Clinical Medicine, University of Bergen, Bergen, Norway; 2 National Centre for Ultrasound in Gastroenterology, Haukeland University Hospital, Bergen, Norway; 3 Norwegian PSC Research Center, Department of Transplantation Medicine, Division of Cancer Medicine, Surgery, Inflammatory Diseases and Transplantation, Oslo University Hospital, Oslo, Norway; Mayo Clinic Minnesota, UNITED STATES

## Abstract

This study aimed to assess and validate the repeatability and agreement of quantitative elastography of novel shear wave methods on four individual tissue-mimicking liver fibrosis phantoms with different known Young’s modulus. We used GE Logiq E9 2D-SWE, Philips iU22 ARFI (pSWE), Samsung TS80A SWE (pSWE), Hitachi Ascendus (SWM) and Transient Elastography (TE). Two individual investigators performed all measurements non-continued and in parallel. The methods were evaluated for inter- and intraobserver variability by intraclass correlation, coefficient of variation and limits of agreement using the median elastography value. All systems used in this study provided high repeatability in quantitative measurements in a liver fibrosis phantom and excellent inter- and intraclass correlations. All four elastography platforms showed excellent intra-and interobserver agreement (interclass correlation 0.981–1.000 and intraclass correlation 0.987–1.000) and no significant difference in mean elasticity measurements for all systems, except for TE on phantom 4. All four liver fibrosis phantoms could be differentiated by quantitative elastography, by all platforms (p<0.001). In the Bland-Altman analysis the differences in measurements were larger for the phantoms with higher Young’s modulus. All platforms had a coefficient of variation in the range 0.00–0.21 for all four phantoms, equivalent to low variance and high repeatability.

## Introduction

Elastography is a non-invasive imaging technique that aims to assess tissue elasticity in several organs through quantitative or semi-quantitative measurements. In the last years, several manufacturers have introduced new elastography methods, offering shear-wave based elasticity mapping or measurement integrated in high-end scanners. The methods are aimed to be used as a clinical tool in several fields of medicine, however the use of shear wave elastography (SWE) methods has predominantly focused on application in chronic liver diseases. The elastography methods that are implemented vary by technique, reported parameter, application and are not standardized to a common use. The different manufacturers apply propriety patented calculation modes, which might result in different values. [1, 2] This has been addressed in previous studies where liver elasticity has been assessed, and several papers have confirmed that the different technologies have different cut-off values. [1, 3, 4] However, it is important that comparative studies address the repeatability and agreement of the emerging technologies in vitro as well as in vivo in healthy and non-healthy patients. So far there is not enough scientific evidence in the literature to validate the most recent technologies.

All elastography methods are based on that the tissue elasticity is measured by Young’s modulus as pressure in kilopascals (kPa). The relationship between the applied stress and resulting strain is defined by Young’s modulus and quantifies tissue elasticity. This means that the harder the tissue elasticity is, the higher Young’s modulus (elasticity) will be.

The SWE methods use an acoustic pulse to create shear waves that travel perpendicularly to, and much slower than the longitudinal ultrasound (US) waves, making it possible to track and measure them within a limited distance. [5, 6] The velocity of the propagating shear waves is faster in harder than in softer tissue, making it a useful method in the evaluation of soft tissue. The main elastography technologies can be divided into strain imaging, shear wave speed measurement and shear wave speed imaging (2D-SWE). The technologies differ by the type of force applied, the visual representation of tissue elasticity and possibility to perform quantitative assessment of recorded tissue elasticity. [7] The elasticity measurements, using SWE or 2D SWE, may be expressed as either shear wave velocity (m/s) or Young’s modulus (kPa).

Most SWE methods integrated into US scanners provide real-time visualization (Brightness mode/B-mode) allowing the examiner to position the specific area of interest for elasticity measurements. This is of great clinical value as it gives the ability to evaluate the liver tissue, and perform elastography measurements whilst avoiding vessels and choosing the region of interest at the right depth from the liver capsule. Elasticity itself is often not visualized (Point shear wave elastography; VTQ and ElastPQ); however, some 2D-shear wave elastography (2D-SWE) methods, including GE 2D-SWE and Supersonic SWE, offer real time visualization of elasticity by a color map within the measurement area and a numerical calculation of shear wave speeds or elasticity. One exception is TE, which was one of the first elastography technologies available. [8–10] While well validated in the literature, TE lacks ultrasound visualization and cannot be applied in patients with perihepatic ascites. [7]

The aim of this study was to compare and assess the agreement and repeatability of three novel elastography technologies and compare their results to one established shear wave method on liver fibrosis phantoms.

## Material and methods

### Study design

We used five different elastography systems, which were all commercially available and approved for medical use in diagnostic ultrasound. The systems reported the tissue elasticity in meters per second (m/s) or kilopascals (kPa), as Young’s modulus. Two individual observers (A.M. and A.B.M.) obtained data from all elastography methods individually, blinded to each other’s results. Each observer (A and B) performed free-hand scanning of the four, separate, tissue-mimicking phantoms and made ten separate measurements of each phantom using the same elastography imaging settings. Both observers had more than 2 years’ experience in ultrasound and elastography. Only one observer was certified for Fibroscan. The curvilinear probes were applied for imaging and elastography on the ultrasound scanners, whilst the M-probe was applied for TE. The region of interest (ROI) was standardized; for 2D-SWE 1 cm circle, for S-Shearwave Elastography and Acoustic Radiation Force Impulse (ARFI) a standardized box 1x0.5 cm and for Shear Weave Measurement (SWM) 1x1.5 cm and fixed in size. The ROI was placed 2–3 cm under the liver fibrosis phantom surface. The elastography systems were evaluated for inter- and intraobserver variability by, coefficient of variation, interclass correlation and limits of agreement using the median value. Each image was recorded to the hard drive of the scanners and stored to an external storage device. Software versions and default settings are provided in the Appendix.

### The objects of examination

The object of examination were liver fibrosis phantoms manufactured by Computerized Imaging Reference Systems (CIRS Inc. Virginia, USA). The model 039 consisted of four separate phantoms of varying stiffness (Table 1). Each phantom was 10 cm deep and made with Zerdine®, a patented synthetic polymer, housed in a 14 cm tall and 11.6 cm wide cylinder with a Saran-based scan surface and a scanning well. The phantom was compatible with the ultrasound shear wave modalities including Fibroscan Transient Elastography and ARFI. It had standard configuration with the following nominal acoustic properties: Attenuation: 0.5dB/cm/MHz, Contrast: 0 dB with respect to CIRS liver reference. The actual acoustic and mechanical properties of each phantom had been batch tested by the manufacturer by an external method, and the measured and calculated values are provided in Table 1. Similar cylindrical Zerdine phantoms from CIRS have been determined to be adequately homogeneous based on testing performed by the Nightingale Laboratory at Duke University and QIBA (Dept. of Biomedical Engineering). [11, 12]

**Table 1:** Expected measurements and acoustic properties for the liver fibrosis phantoms represented with ±5% SD.

Phantom	Young’s modulus (kPa)		Density (g/cm^3^)	Speed of sound (m/s)	Expected shear wave velocity (m/s)
**1**	2.7 ± 0.14		1.03	1533	1.62 ± 0.08
**2**	11.5 ± 0.57		1.03	1536	3.34 ± 0.17
**3**	24.8 ± 1,24		1.03	1531	4.91 ± 0.25
**4**	46.3 ± 2.32		1.03	1530	6.70 ± 0.34

### Elasticity imaging and SWE platforms applied

GE 2D-Shear wave elastography (SWE)

**Fig 1:**
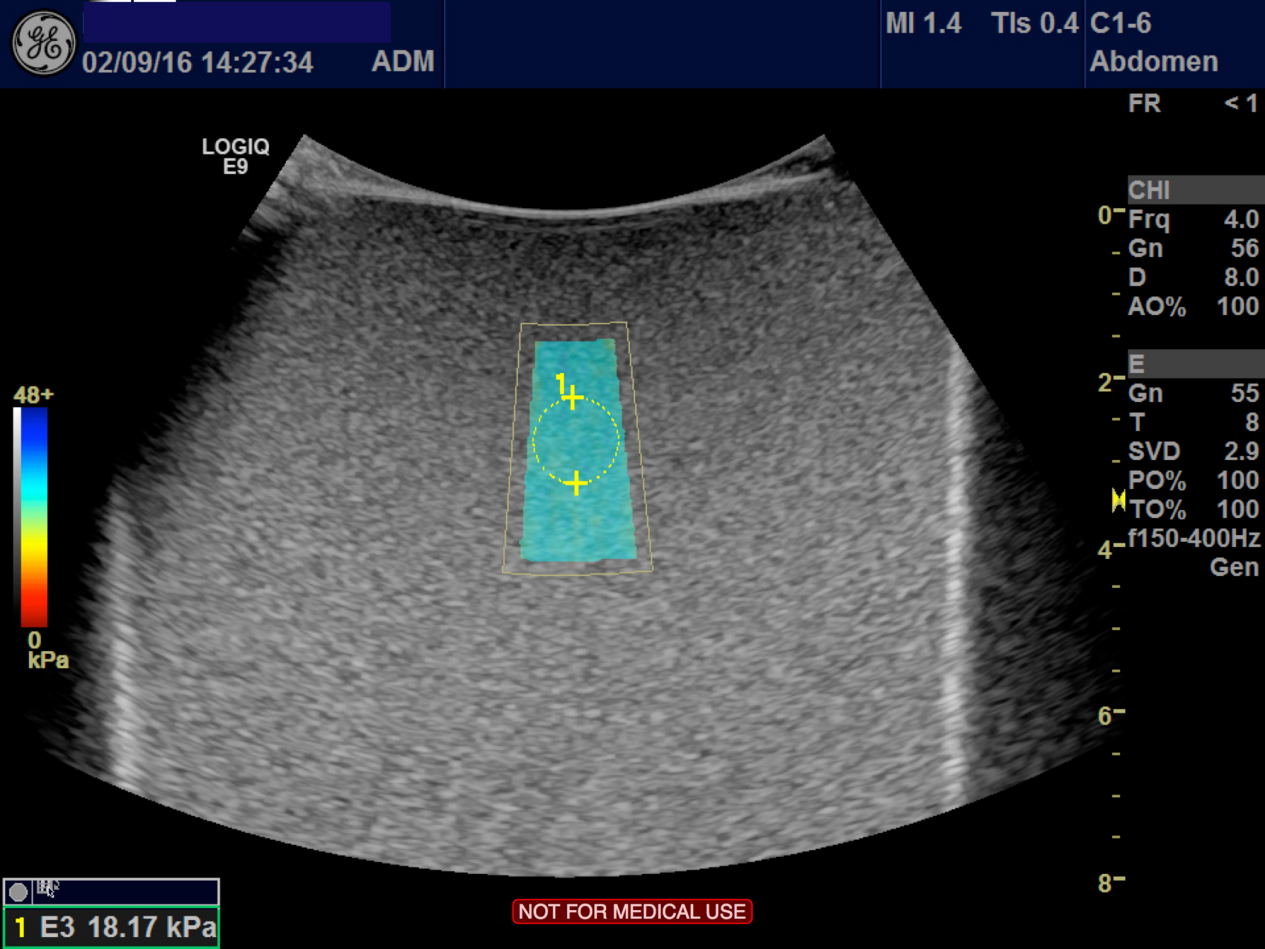
GE 2D-SWE. The figure illustrates the method of 2D-SWE by GE performed on liver fibrosis phantom 3 with Young’s modulus 24.8 kPa ±5%. The color box (centre) represents the elastogram, and the circle represents the ROI where the elasticity measurement is acquired. The blue color indicates harder tissue, as semi-quantitatively presented by the color scale to the left.

The elastography method of 2D-SWE was applied from the system of LOGIQ E9 (GE Healthcare, Milwaukee, Wisconsin, USA) Version 2.0, using the C1-6 probe. The method generates shear wave velocity through an acoustic push pulse, creating a color mapped elastogram. The color indicated the stiffness of the tissue, where red was soft and blue hard. Within the color map, the operator could place a region of interest (ROI) and adjust the size of the ROI. After placing the ROI, under default scanner settings, the elasticity measurements were automatically acquired by the system. (Fig 1) In our study we standardized the size of our ROI to 1 cm and the measurement was obtained at least 2 cm inferior of the liver fibrosis phantom surface. [13] The measurements were expressed in kPa.

Samsung S-Shearwave Elastography (S-SWE)

**Fig 2:**
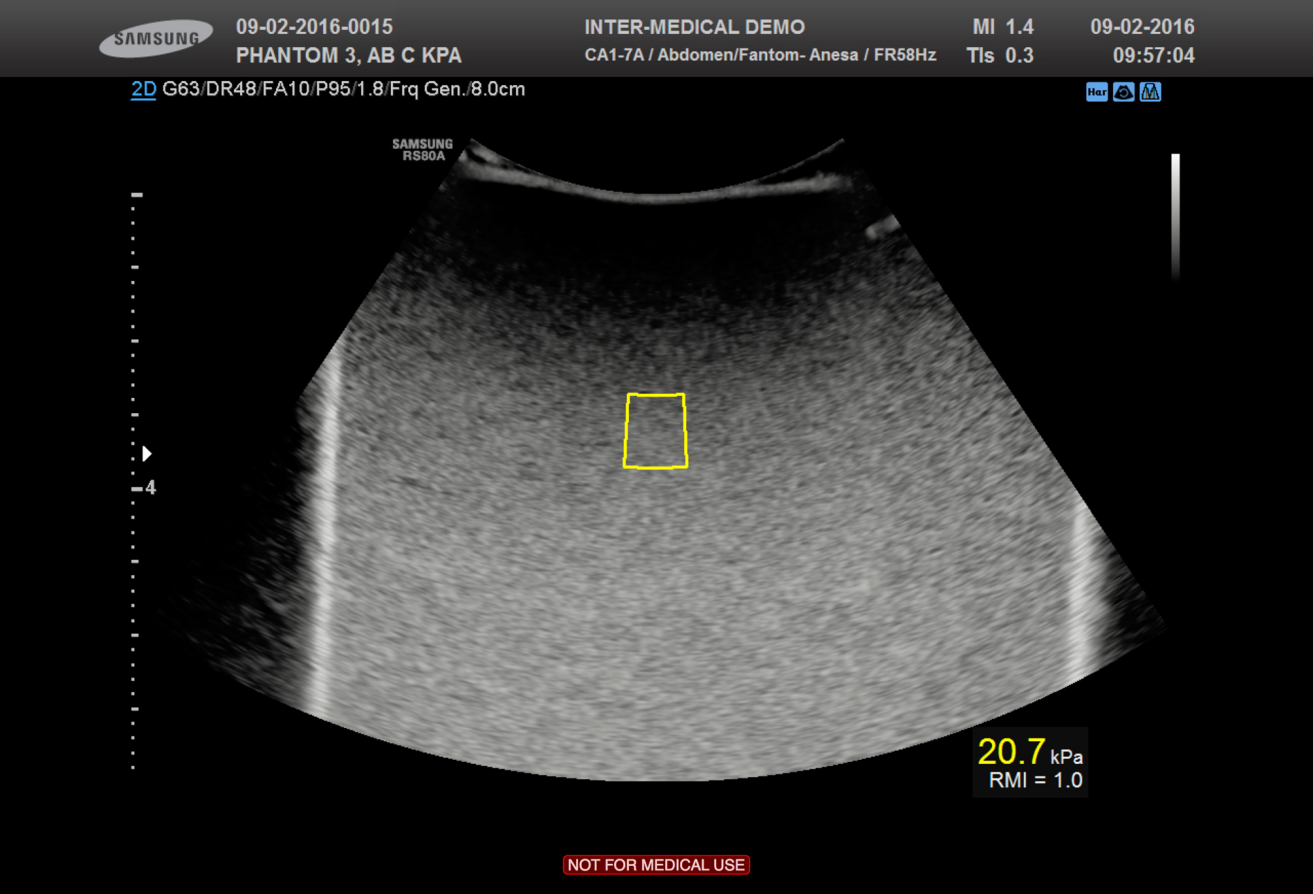
Samsung (S-SWE). Samsung S-Shearwave Elastography assessed on liver fibrosis phantom 3. The yellow box (centre) represents the shear-wave measurement area. ROI and the RMI (Reliability Measurement Index) is expressed below the obtained elasticity measurement of 20.7 kPa.

Using the RS80A with Prestige ultrasound equipment (Samsung Medison Co. Ltd., Seoul, Korea) we assessed the S-Shear wave Elastography (Version 2.0). Within the brightness mode (B-mode) window, using default scanner settings, the ROI could be placed freely and had a fixed height of 1 cm. (Fig 2) The width was automatically adjusted depending of the measurement depth. If the ROI was placed in an area where measurements could not be obtained, for example at 7 cm depth, the color of the box changed to orange, symbolizing an invalid position. We placed the ROI at least 2 cm inferior of the phantom surface. The measurements were expressed in m/s and kPa simultaneously. The method had a unique performance index, “Reliability Measurement Index” (RMI), which is calculated by the weighted sum of the residual of the wave equation and the magnitude of the shear wave. [14] RMI ranging from 0.0–1.0, where 0.4 or higher is considered as acceptable whilst 1.0 is considered a very high value of RMI, and strongly correlates with reproducible measurements, according to the manufacturer. The proposed index is utilized to filter out unreliable measurements and result in performance improvement of shear wave elastography.

Hitachi Shear Wave Measurement (SWM)

**Fig 3:**
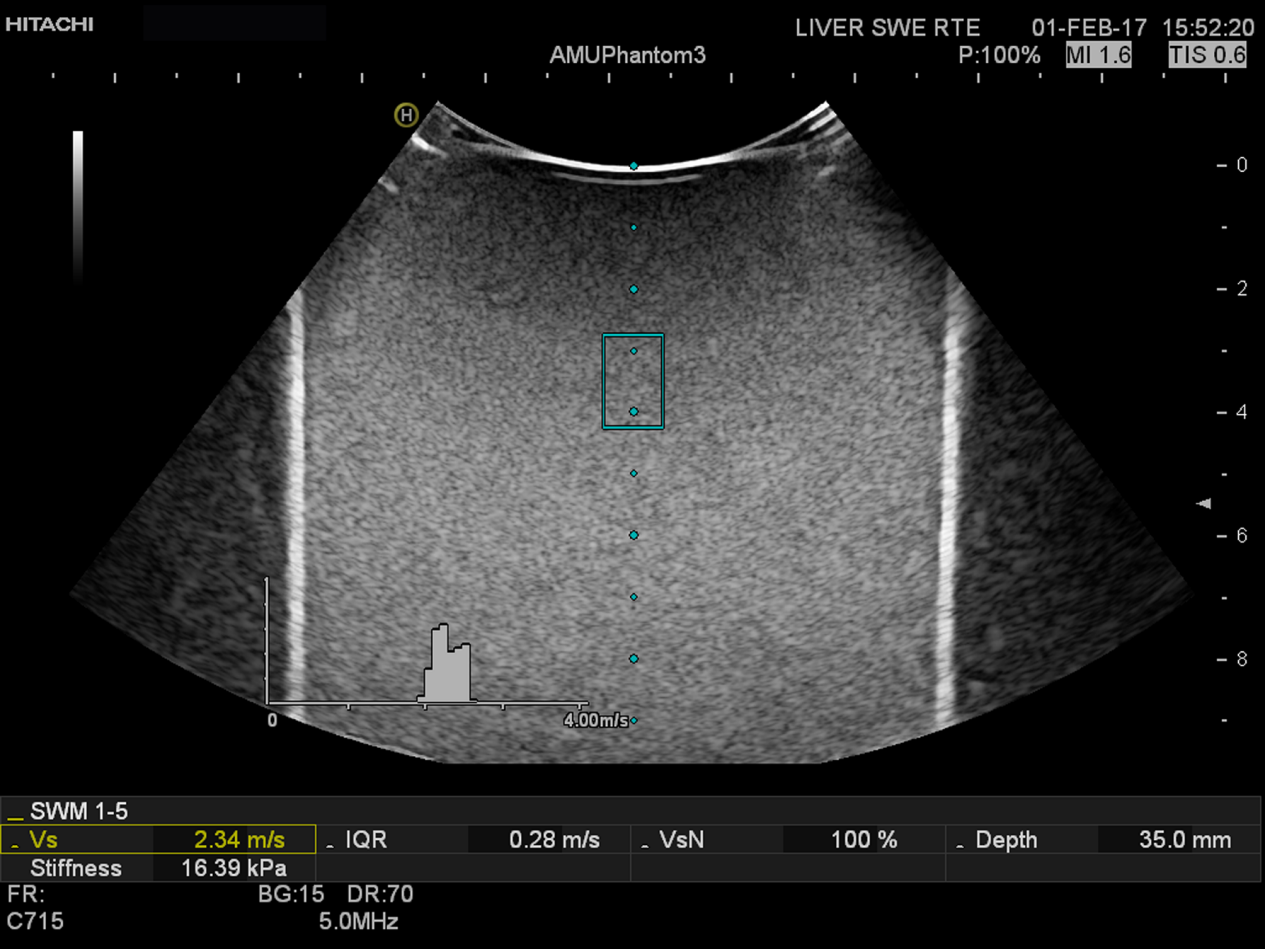
Hitachi (SWM). Hitachi SWM was applied on liver fibrosis phantom 3. The ROI is represented by the blue box (centre). The shear wave velocity measurements are presented in the histogram, and the median is given as Vs in m/s. Stiffness is based on this value expressed in kPa as well as the IQR (m/s), VsN (Reliability Index for shear wave velocity measurement) and the depth of the sample.

Using a Hitachi HI VISION Ascendus (Hitachi Medical corporation, Tokyo, Japan) scanner SWM was applied using the EUP-C715 probe (1-5MHz). Within one SWM measurement several push track sequences are delivered and the SWM samples the shear wave velocity in multiple positions, at different depths, inside the ROI. This is automatically repeated within a short time (<1second). Per acquired SWM, the system displays a histogram and measurement overview. The distribution of the multiple velocity measurements (Vs) were displayed in a histogram, the IQR, depth of sample, the median of Vs in m/s and transformed to kPa. (Fig 3) The method has a built-in feature, the VsN, which is a reliability index of the Vs values acquired per measurement and functions as a quality indicator, and ranges from 0–100%. [15] Ten repeated acquisitions were made, using default scanner settings, and the results were given in m/s. The elasticity was also provided in kPa, by calculation of Young’s modulus, configured by Hitachi’s application specialist.

Philips point shear-wave elastography (pSWE)

**Fig 4:**
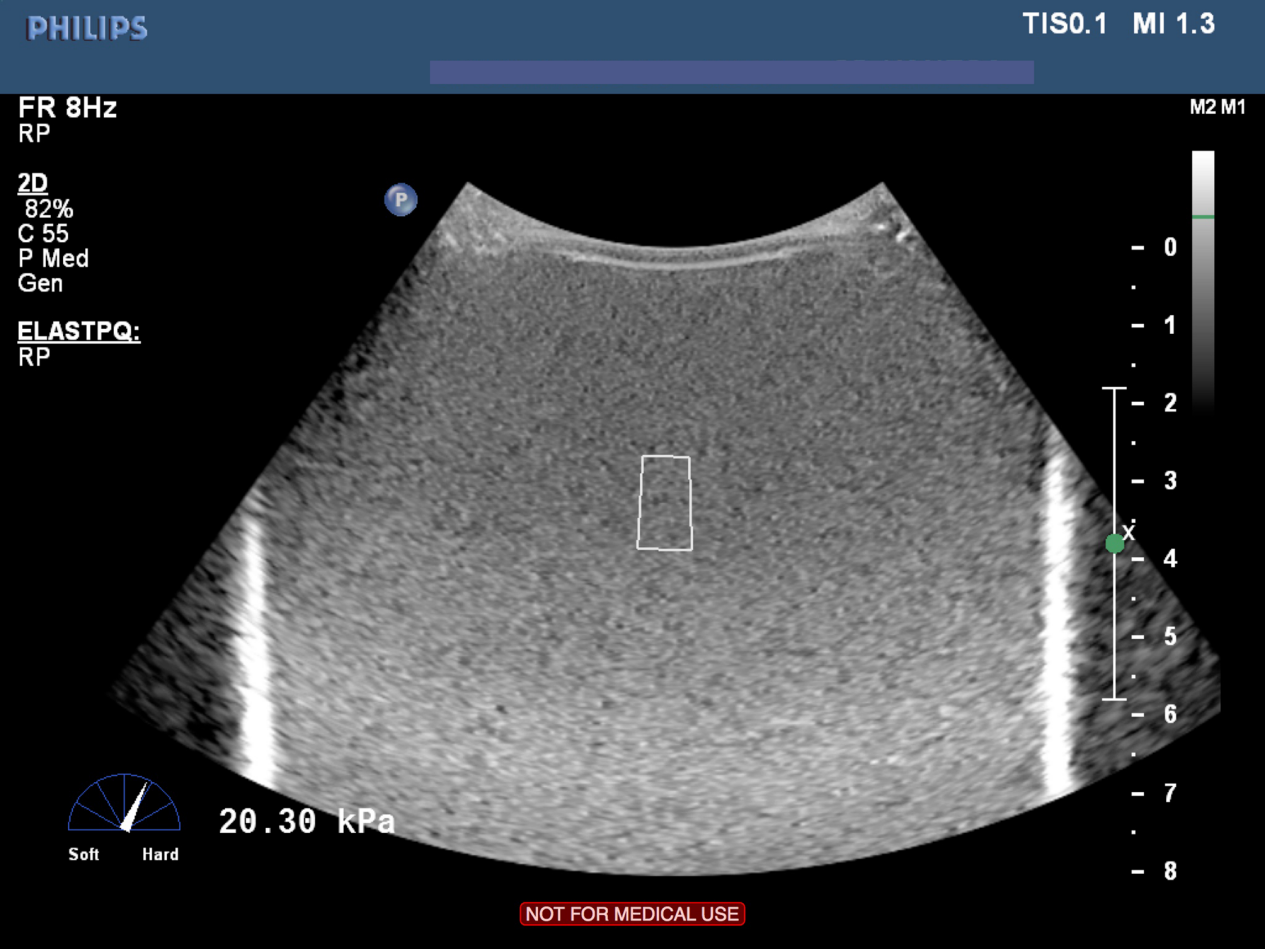
Philips ARFI (pSWE). Philips pSWE elastography is applied on liver fibrosis phantom 3. The shear wave measurement area is represented by the white box (center). The stiffness is shown in kPa on the left together with the unnumbered scale indicating the stiffness of the tissue, here shown towards hard.

Using a Philips iU22 (Eindhoven, Netherlands) a point shear wave elastography platform, also known as ARFI quantification, was applied using a C5-1 probe. The method is based on a quantitative measurement of tissue elasticity, as the ultrasound probe produces a dynamic force that is applied through focused radiation force impulse. This generates shear waves that propagate perpendicularly to the push pulse through the tissue, across the ROI where the propagation of shear wave velocity is measured [2, 16]. The measurements were obtained, using default scanner settings, applying minimum pressure to the phantom surface whilst holding the probe still. The ROI, which was standardized and had a fixed area of 0,5x1 cm, was placed within the field of view obtaining an elasticity measurement. (Fig 4) 10 individual measurements were repeated, and the results were displayed as a median and mean with standard deviation (SD) of 10 measurements. The elasticity was expressed in kPa.

Fibroscan (Transient elastography, TE)

**Fig 5:**
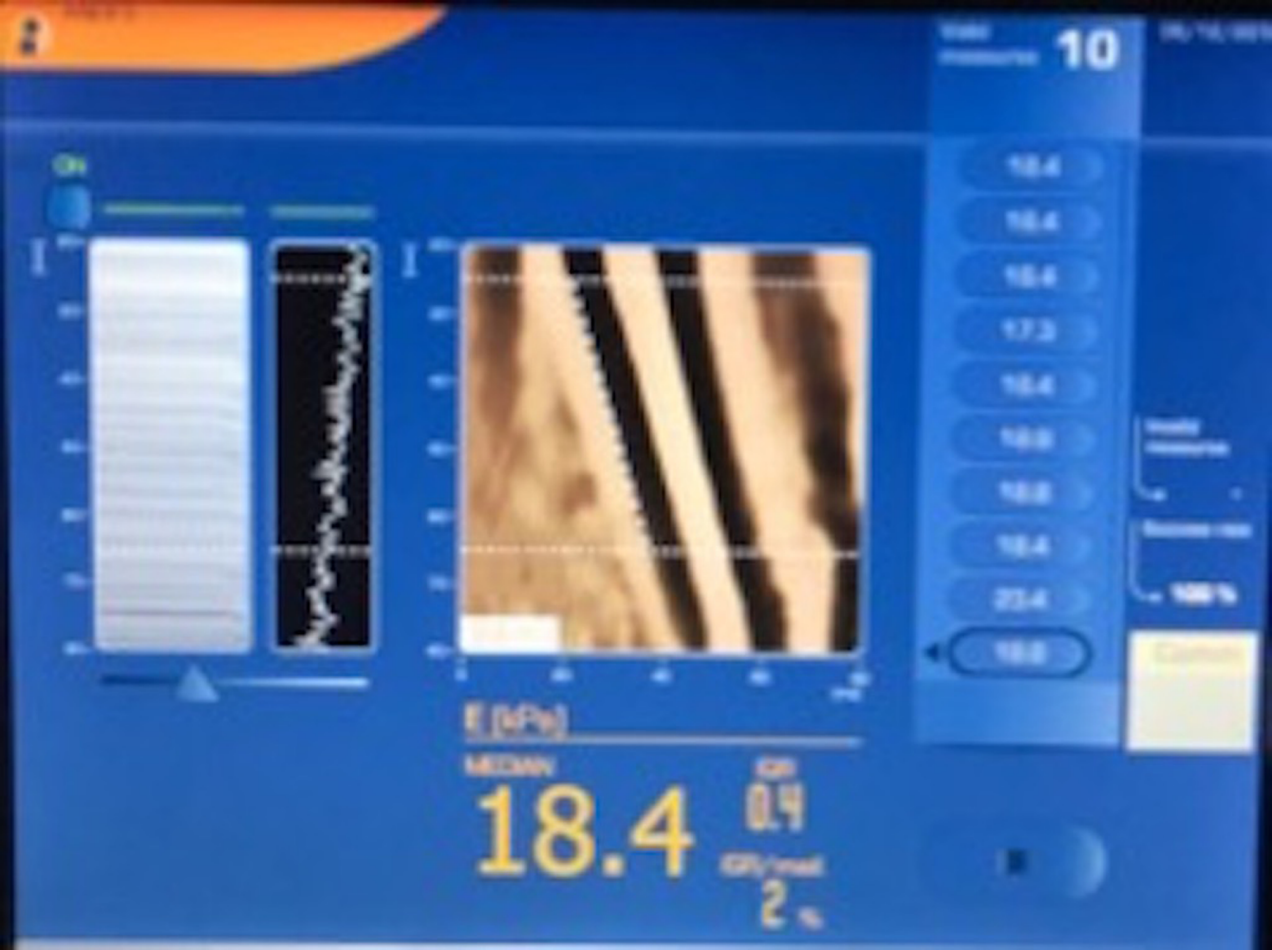
Fibroscan (Transient Elastography, TE). This figure illustrates the assessment of transient elastography on liver phantom 3. 10 valid elastography measurements are listed on the right side, where also success rate and invalid measurements are reported. The IQR/median is used as a quality parameter, and aimed to be below 30% while obtaining a success rate of at least 60%.

Applying Fibroscan® 204 (EchoSens, Paris, France), we used the standard M-probe with a transducer frequency of 3.5 MHz on all four phantoms. The probe generates a vibration with 50 Hz frequency and 2 mm amplitude, which induces a shear wave propagation. The velocity of the shear wave is directly calculated by the device and the results are expressed in kilopascals (kPa) without B-mode. [2, 17] Valid measurements were performed. Both observers aimed to fulfill all quality parameters when performing the measurements. (Fig 5) The device displayed median value of ten measurements, number of failed measurements, the IQR and IQR/median. Reliable measurements were defined by the producer: a measurement success rate (SR) of ≥60% and IQR of <30%. [17, 18]

### Statistical analysis

The statistical analysis was performed using SPSS, Version 24.0, IBM Statistics (Armon, New York, NY, USA). Descriptive statistics and one-way analysis of variance was used to analyze the data. The measurements are represented as median values with min-max for 10 measurements of each phantom, and for each system. The interquartile range (IQR) and the dispersion of the measurements is represented in the boxplots. A higher box (IQR) represents a larger spread in measurements, and represents the data between the 25th and 75th percentile, essentially the range of the middle 50% of the data. Reliable measurements were defined as: median value of 10 valid LS measurements with a success rate (ratio of the number of successful acquisitions divided by the total number of acquisitions) ≥60% and an interquartile range interval < 30%. IQR/Median (%) is illustrated in the bar charts, and was calculated for both observers individually as well as together and for all systems. [17, 18] We calculated the coefficient of variation (CV) of the intraobserver variability, which is the standard deviation (SD) divided by the mean value. Inter-class correlation coefficients (ICC) were calculated to present the interobserver reliability; ICC near 1.00 indicated high reliability. One-way ANOVA and Tukey’s test was used to test the overall significance, and p <0.01 was chosen as level of significance because we performed multiple testing by platforms and phantoms. Inter-observer agreement was assessed by correlation plots using Pearson’s coefficient of correlation (r). Limits of agreement were assessed to discover differences between individual measurements for each method. [19, 20]

## Results

**Fig 6:**
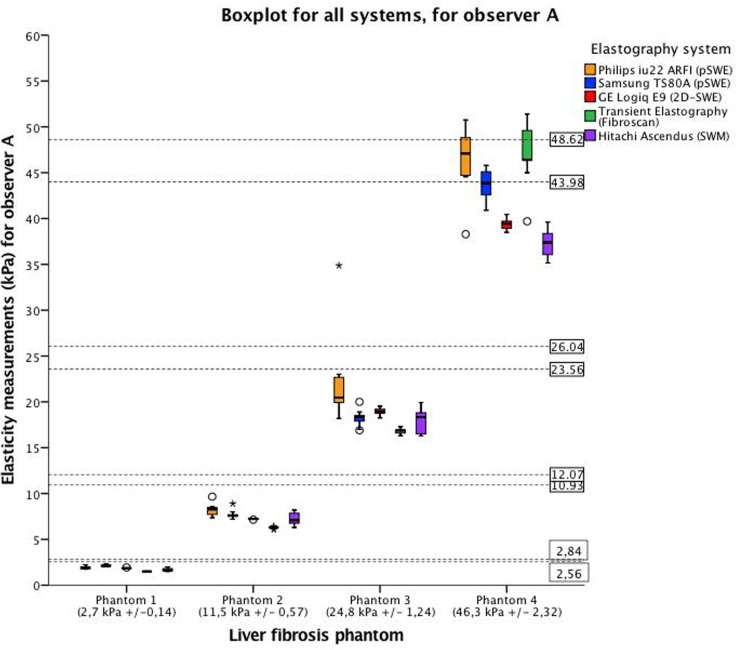
Variation in elasticity measurements for all systems for observer A. The boxplot displays the median and the interquartile range, whiskers represent the 90th percentile of the measured elasticity by observer A for the four phantoms. The height of the box represents the measurement variability of the single observer for each of the phantoms. The horizontal axis represents the four phantoms with increasing stiffness; phantom 1 (2,7 ±0,14 kPa), phantom 2 (11,5 ± 0,57 kPa), phantom 3 (24,8±1,24 kPa) and phantom 4 (46,3 ± 2,32 kPa). The range each phantom stiffness is presented by the dotted lines within the figure. The vertical axis represents elasticity measurements (kPa) obtained by observer A. The colors represent the systems applied in the study: yellow, Philips iU22 (pSWE); blue, Samsung TS80A (pSWE); red, GE E9 (2D-SWE); green, Fibroscan (Transient Elastography) and purple, Hitachi Ascendus (SWM).

**Fig 7:**
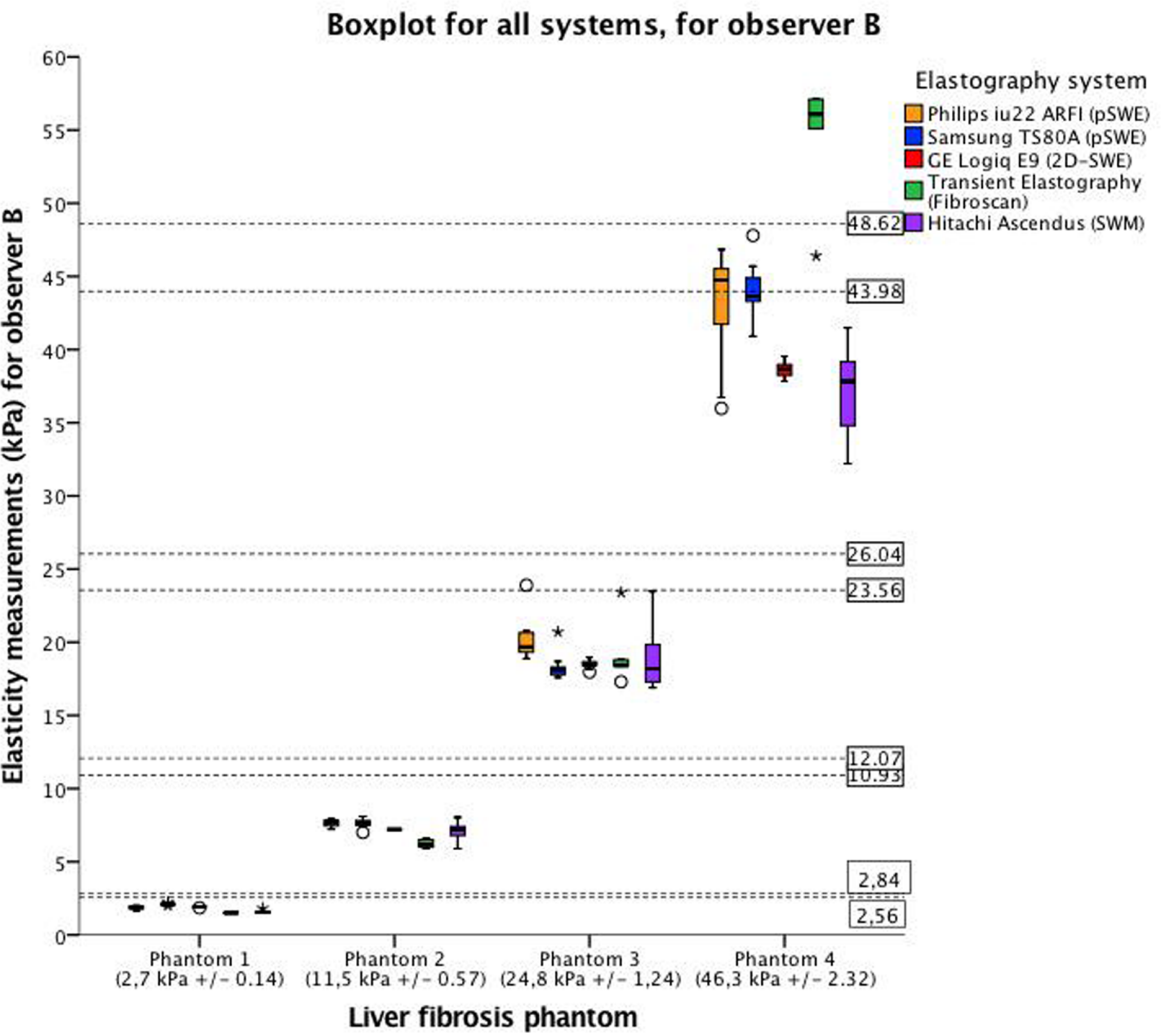
Variation in elasticity measurements for all systems for observer B. The boxplot displays the median and the interquartile range, whiskers represent the 90th percentile of the measured elasticity by observer B for the four phantoms. The height of the box represents the measurement variability of the single observer for each of the phantoms. The horizontal axis represents the four phantoms with increasing stiffness; phantom 1 (2,7 ±0,14 kPa), phantom 2 (11,5 ± 0,57 kPa), phantom 3 (24,8±1,24 kPa) and phantom 4 (46,3 ± 2,32 kPa). The range each phantom stiffness is presented by the dotted lines within the figure. The vertical axis represents elasticity measurements (kPa) obtained by observer B. For color representation, we refer to Fig 6.

**Fig 8:**
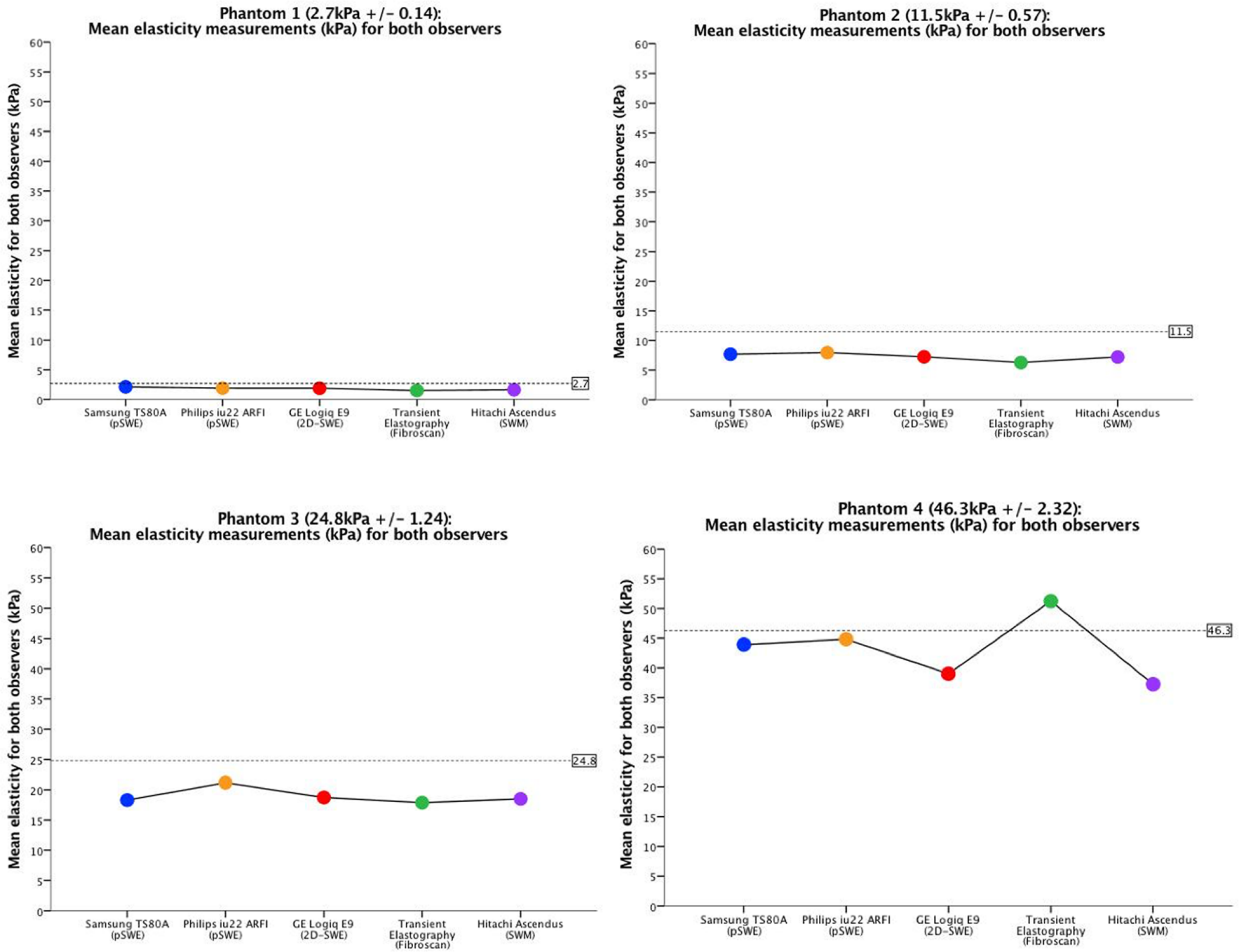
Mean elasticity measurements for both observers. The figure shows the common mean for both observers within each phantom and for all systems. On the horizontal axis, the systems are listed with name, and on the vertical axis the mean elasticity measurements are expressed in kPa. The dotted line within the graph represents the elasticity of the respective phantom, provided by the producer. For color representation, we refer to Fig 6.

All liver fibrosis phantoms (1–4) could be significantly differentiated by all elastography methods (p <0.001) as illustrated by the boxplots in Figs 6 and 7. Figs 6 and 7 shows the measurement variability, interquartile range (IQR) and median values, represented by the vertical distribution of the box, which is illustrated in different colors for the respective systems. All systems showed a low variability for the softer phantoms (1 and 2), compared with the harder phantoms (3 and 4). This is confirmed in the correlation analysis and limits of agreement. When acquiring elasticity measurements (mean for both observers) of phantom 3, Philips ARFI showed higher mean and measurement variability compared to all the other systems. (p<0.001). Both observers obtained higher elasticity measurements with all the shear wave methods than TE for phantom 1–3, and lower for phantom 4. (Fig 8) Furthermore, we could not demonstrate significant difference in elasticity measurements and variability between Samsung, GE, Fibroscan, Hitachi. For phantom 4, differences in mean elasticity measurements between the systems were statistically significant difference (p<0.001), however, Philips and Samsung (p = .869), and GE and Hitachi (p = .355), did not demonstrate significant differences, respectively. CV was in the range 0.00–0.21. Philips ARFI showed the highest CV for phantom 3 (observer A: CV = 0,21). The mean values for each observer and CVs are given in Table 2 and illustrated in Figs 6, 7 and 8.

**Table 2:** Mean and median of measurements for observer A (Mean/Median A) and B (Mean/Median B) in all liver fibrosis phantoms (1–4) and for all systems.

	Philips iU22 XM (ARFI)	Samsung RS80A (SWE)	GE Logiq E9 (2D-SWE)	Hitachi Ascendus (SWM)	Fibroscan (TE)
**Phantom 1**	**2.7 kPa ± 0.14**				
Mean/Median A	1.94/1.93	2.12/2.10	1.86/1.86	1.67/1.61	1.50/1.50
Mean/Median B	1.87/1.89	2.11/2.10	1.91/1.92	1.59/1.56	1.50/1.50
CV A	0.08	0.04	0.02	0.10	0.00
CV B	0.06	0.03	0.01	0.06	0.00
CV AB	*0*.*07*	*0*.*04*	*0*.*02*	*0*.*08*	*0*.*00*
**Phantom 2**	**11.5 kPa ± 0.57**				
Mean/Median A	8.24/8.29	7.71/7.60	7.24/7.24	7.27/7.11	6.28/6.30
Mean/Median B	7.64/7.73	7.63/7.65	7.20/7.21	7.12/7.21	6.25/6.20
CV A	0.08	0.06	0.01	0.09	0.02
CV B	0.03	0.04	0.00	0.08	0.04
CV AB	*0*.*06*	*0*.*05*	*0*.*01*	*0*.*08*	*0*.*03*
**Phantom 3**	**24.8 kPa ± 1.24**				
Mean/Median A	22.18/20.46	18.27/18.40	18.94/18.92	17.97/18.34	16.80/16.90
Mean/Median B	20.17/19.68	18.31/18.15	18.51/18.47	19.01/18.20	18.91/18.40
CV A	**0.21**	0.05	0.02	0.07	0.02
CV B	0.07	0.05	0.02	0.12	0.09
CV AB	0.14	*0*.*05*	*0*.*02*	*0*.*09*	*0*.*05*
**Phantom 4**	**46.3 kPa ± 2.32**				
Mean/Median A	46.64/47.09	43.76/43.85	39.40/39.44	37.31/37.38	47.23/46.40
Mean/Median B	43.03/44.74	44.09/43.65	38.62/38.67	37.22/37.83	55.23/56.10
CV A	0.09	0.04	0.02	0.04	0.07
CV B	0.09	0.04	0.01	0.08	0.06
CV AB	*0*.*09*	*0*.*04*	*0*.*01*	*0*.*06*	*0*.*07*

Coefficient of variation for observer A (CV A) and B (CV B) and for both observers (CV AB).

**Fig 9:**
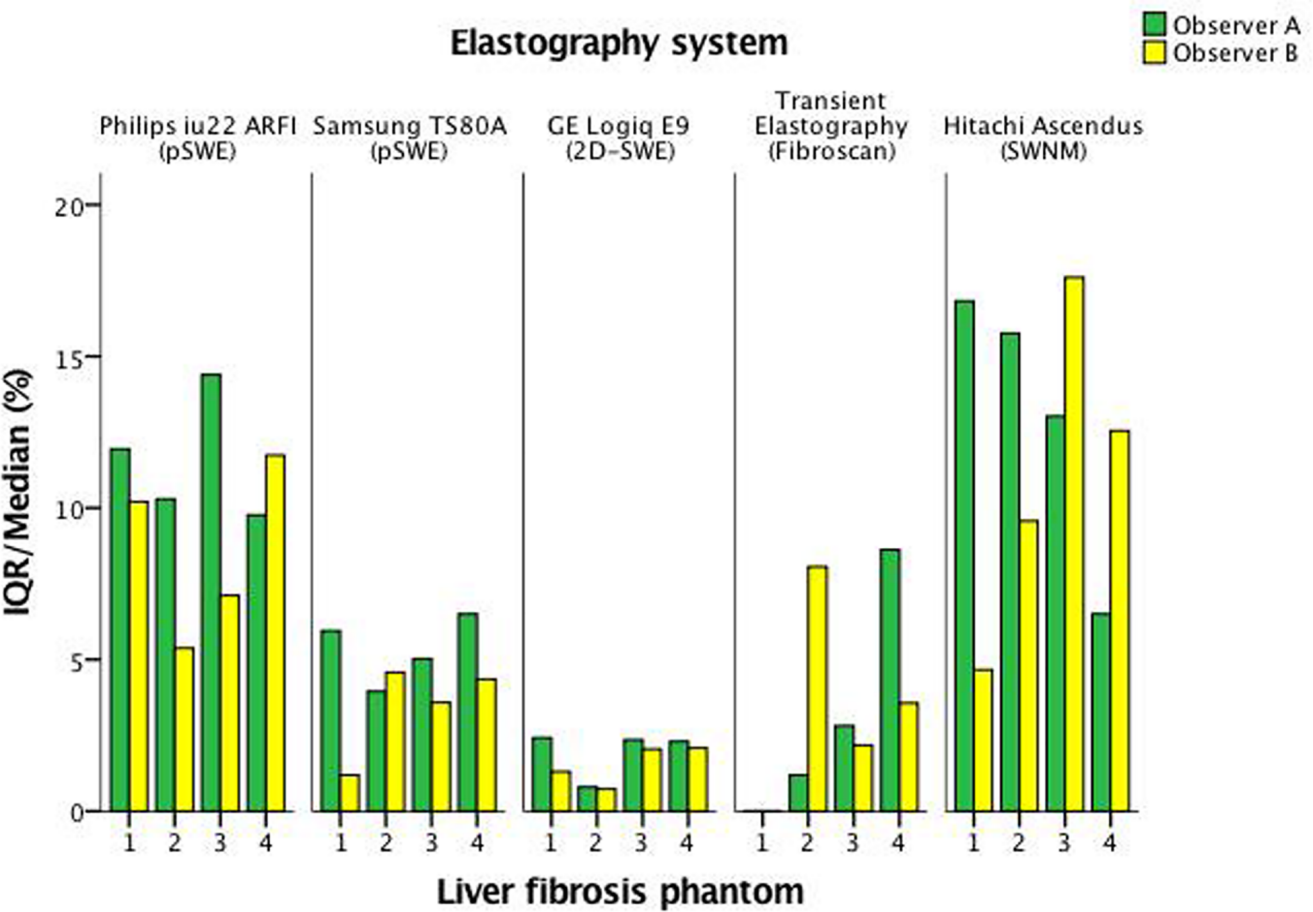
IQR/Median (%) for all systems and both observers. IQR/Median (%) is presented on the vertical axis for observer A (green) and B (yellow). The phantoms 1–4 are numbered on the horizontal axis.

**Fig 10:**
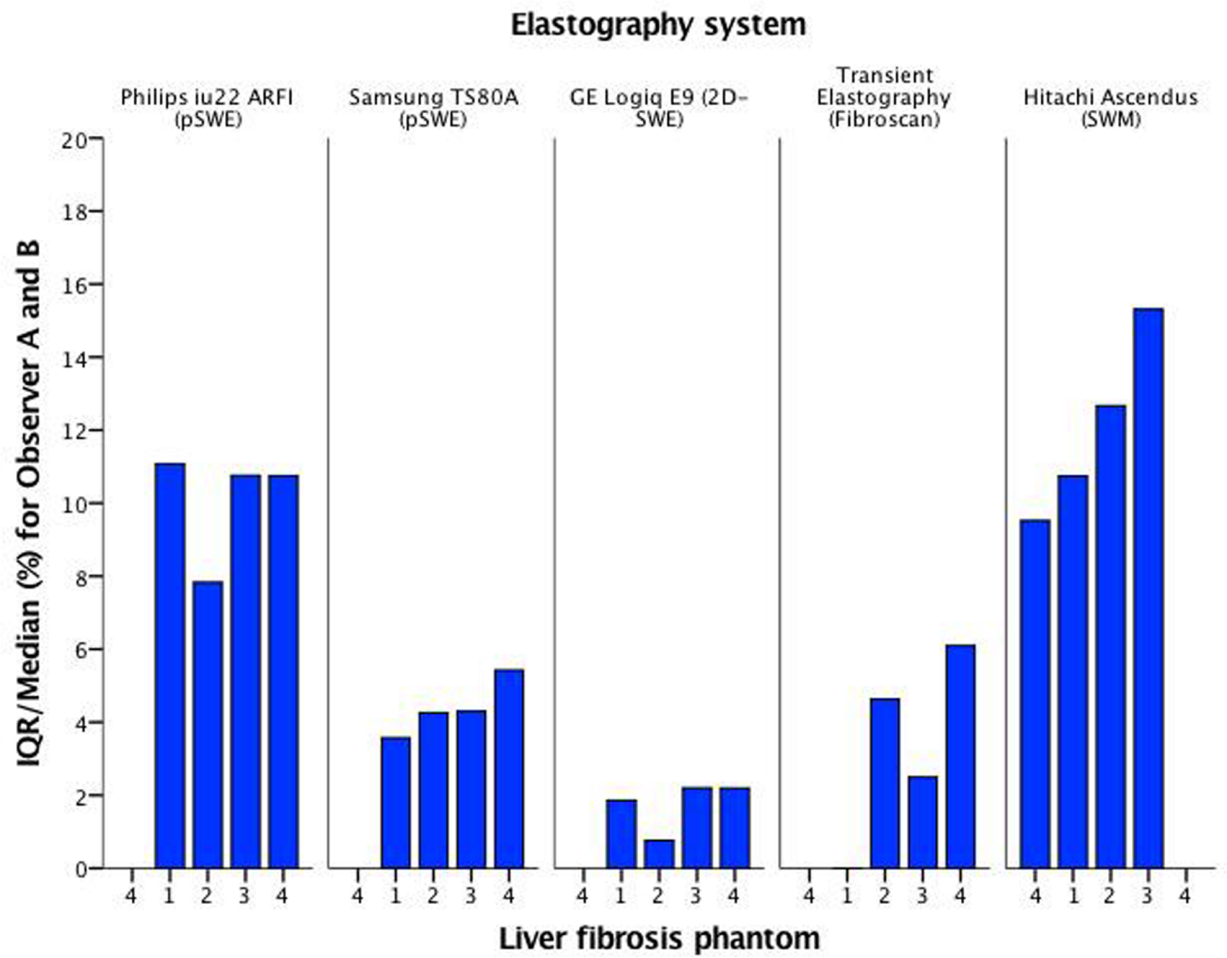
IQR/Median (%) for all systems and both observers. IQR/Median (%) is presented on the vertical axis for both observers (blue). The phantoms 1–4 are numbered on the horizontal axis.

All systems had reliable measurements and an IQR/median <30% when applied in vitro (Figs 9 and 10). GE 2D-SWE and Samsung RS80A (pSWE) showed the lowest variation for all phantoms and both observers individually. Transient elastography did not show any variation for the softest phantom for either of the observers, whilst Hitachi (SWM) and Philips (sSWE) demonstrated slightly higher variation for all phantoms and both observers.

Overall, there was no significant difference between observer A and B’s mean elasticity measurements, for most of the systems and all phantoms (1–4) (p = 0.043–1.000). However, there was a significant difference between TE in phantom 4 (p<0.00), as shown in Table 3.

**Table 3:** Level of significance for elasticity measurements between observer A and B, for all systems.

	Philips iU22 XM (ARFI)	Samsung RS80A (SWE)	GE Logiq E9 (2D-SWE)	Hitachi Ascendus (SWM)	Fibroscan (TE)
**Phantom 1**	**2.7 kPa ± 0.14**				
Observer A Mean **± SD**	1.9±0.2	2.1± 0.1	1.9±0.03	1.7±.2	1.5±0
Observer B Mean **± SD**	1.9±0.1	2.1±0.1	1.9±0.03	1.6±0.1	1.5±0
P-value	.686	1.000	.965	.670	1.000
**Phantom 2**	**11.5 kPa ± 0.57**				
Observer A Mean **± SD**	8.2±0.7	7.7±0.5	7.2±0.04	7.3±0.6	6.3±0.1
Observer B Mean **± SD**	7.6±0.2	7.6±0.3	7.2±0.03	7.1±0.6	6.3±0.3
P-value	0.043	1.000	1.000	.998	1.000
**Phantom 3**	**24.8 kPa ± 1.24**				
Observer A Mean **± SD**	22.2±4.7	18.3±0.9	18.9±0.3	18.0±0.9	16.8±0.4
Observer B Mean **± SD**	20.2±1.5	18.3±0.9	18.5±0.3	19.0±2.3	18.9±1.6
P-value	.352	1.000	1.000	.965	.288
**Phantom 4**	**46.3 kPa ± 2.32**				
Observer A Mean **± SD**	46.6±3.7	43.8±1.6	39.4±0.6	37.3±1.4	47.2±3.5
Observer B Mean **± SD**	43.0±3.9	44.1±1.8	38.6±0.5	37.2±2.9	55.2±3.3
P-value*	0.078	1.000	1.000	1.000	<0.00

*P-value >0.05 indicates that there was no significant difference in mean of elasticity measurements between the observers

**Fig 11:**
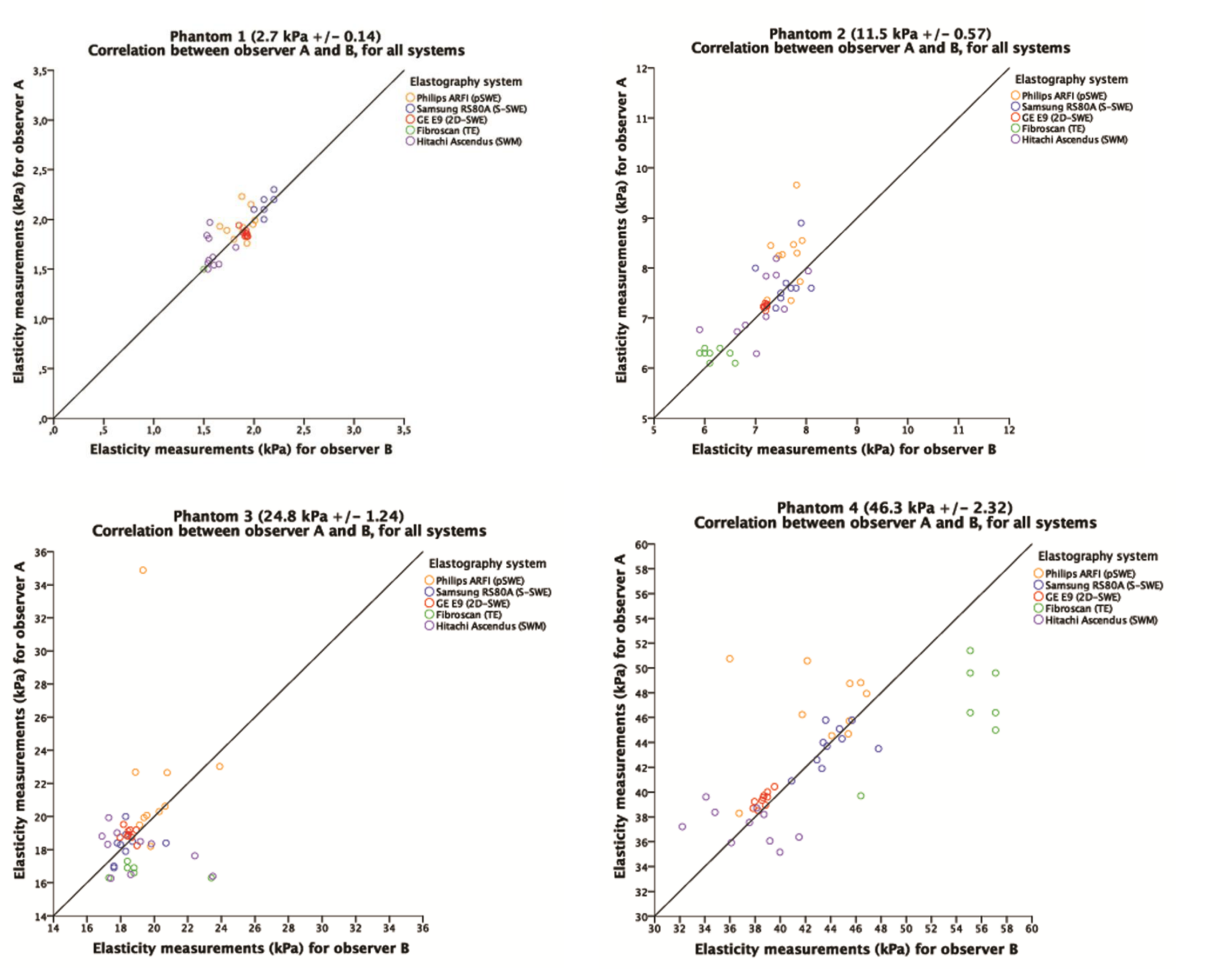
Correlation between observers in measurement of each phantom for all systems. The horizontal and vertical axes represent measurements by observer B and A, respectively. The unit measured is kilopascals (kPa). The line in the graph represents the line of unity. For color representation, we refer to Fig 6.

The correlation of all systems combined, was excellent (r = 0.985). (Fig 11) All systems used in this study provided a high repeatability in quantitative measurements for all liver fibrosis phantoms and excellent correlation between the two observers (Fig 11). Interobservation correlation was 0.981–1 and intraobservation correlation 0.987–1. (Table 3) For all systems, except Philips (p = 0.009), no significant difference in correlation was seen between the observers (p = 0,157–0.660). Pearson’s coefficient of correlation was excellent and in the range of r = 0.981–1.000.

**Fig 12:**
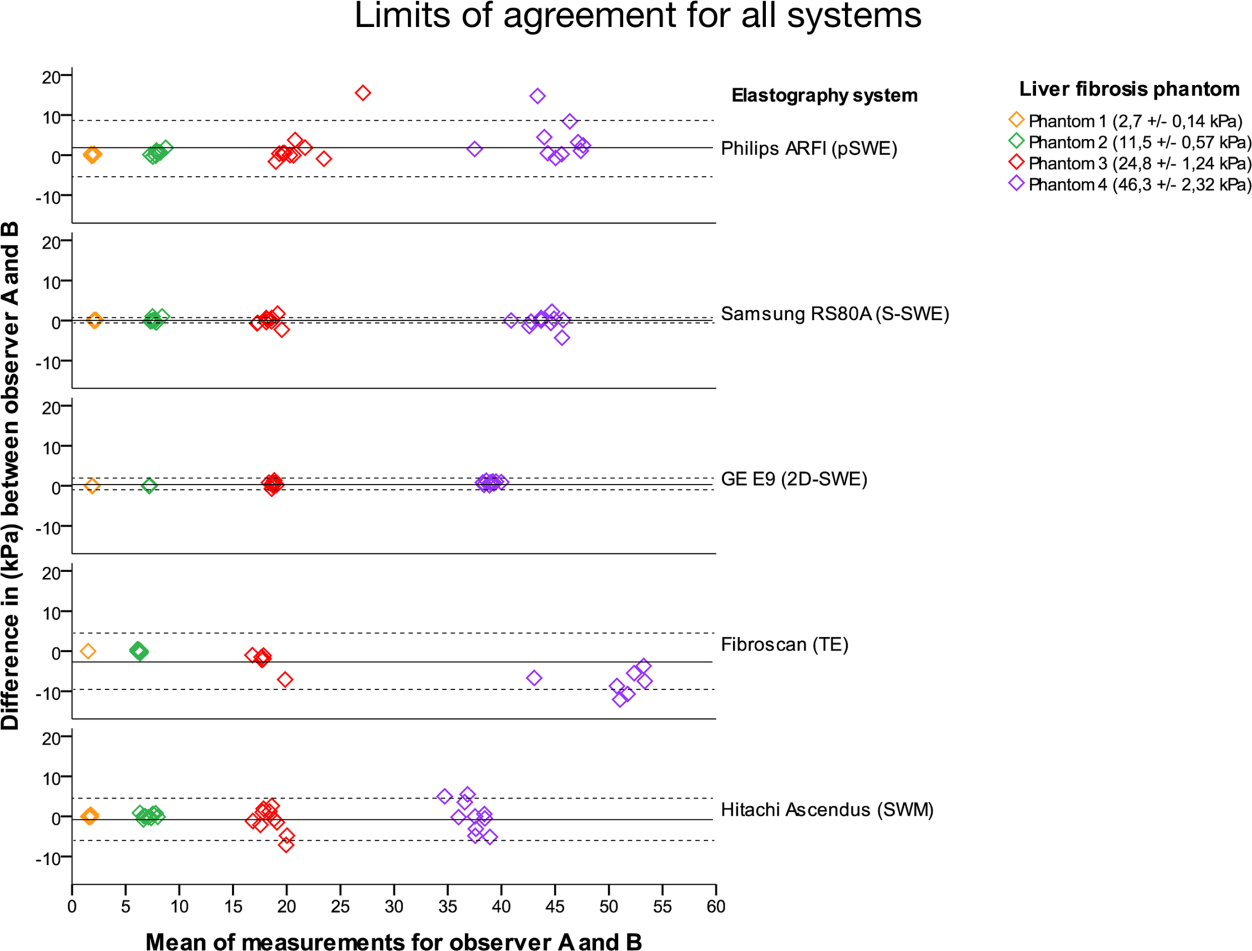
Limits of agreement. The elastography system used are identified on the right side of the graph. The phantoms are identified by color, and represented in the upper right corner. The colors represent the four phantoms with increasing stiffness; yellow as phantom 1 (2,7 ± 0,14 kPa), green as phantom 2 (11,5 ± 0,57 kPa), red as phantom 3 (24,8 ± 1,24 kPa) and purple as phantom 4 (46,3 ± 2,32 kPa). The horizontal axis represents the common mean value of all measurements in both observers while the vertical axis represents the difference between individual measurements and this common mean (kPa), displaying the variability of measurements for the four phantoms. The black line within each system represents the common mean value, the dotted lines represent 95% Confidence Interval. A mean value close to 0 on the vertical axis means that the two observers apply the measurement scale without bias. If it deviates from 0, one of the observers tend to measure higher or lower values systematically compared to the other observer.

The reliability of measurements was demonstrated by the limits of agreement method, based on the difference from a common mean in measurements by observer A and B. Larger deviations of the mean from 0 reflect larger differences between observers, indicating observer bias. For intraobserver variation, we found a higher measurement variability for harder phantoms (phantom 3 and 4) than for the softer (1 and 2). In our study the deviation from the mean was limited for all methods, although all methods illustrated a larger spread in measurements for the harder phantoms (Fig 12).

## Discussion

The expanding spectrum of novel ultrasound elastography techniques demands comparative studies that address the agreement and repeatability of the emerging technologies *in vitro* as well as *in vivo* in healthy and non-healthy patients. The shear wave elastography technologies may vary between the systems from different manufacturers; furthermore, similar techniques applied in different systems may result in different values of shear wave speed measurements based on variations related to frequencies and to the algorithms used to determine tissue properties. [1] Analyses of performance as well as head-to-head comparisons between several novel elastography systems which have been introduced in the market for clinical use over recent years, are still scarce and we believe that the present paper addresses a need in this regard as these systems are already beginning to be employed in the clinical follow-up of patients.

In the present study, we evaluated the use of four different shear wave elastography methods and transient elastography in a head-to-head design, assessing the reliability of measurement acquisition and repeatability *in vitro* using four individual, quality controlled, commercially available liver fibrosis phantoms which represented elasticities ranging from values found in healthy liver tissue to cirrhosis. We found a high degree of repeatability, both for individual observers and between two observers for all the methods, as reported by CV in Table 2 and level of significance in Table 4.

**Table 4:** Inter- and intraclass correlation for both observers, for all systems.

System	Probe		Intraobserver correlation (ICC)	Interobserver correlation A+B (ICC)
**Philips iU22 XM (ARFI)**	C5-1		0.989	0.981
**Samsung RS80A (SWE)**	C5-1		0.999	0.998
**GE Logiq E9** **(2D-SWE)**	C1-5		1	1
**Hitachi Ascendus (SWM)**	C175		0.992	0.985
**Fibroscan (TE)**	M-probe		0.991	0.995

GE 2D-SWE showed the lowest CV (0.00–0.02), with no significant differences in mean elasticity between observers for either soft or hard phantoms (p = .965–1.000). This was the only 2D- SWE method evaluated in this study. The tendency towards higher repeatability compared to the other shear wave methods, may be influenced by a different scanning and measurement procedure compared to the other methods. For GE 2D-SWE, several frames can be acquired within one loop, allowing several measurements in the identical probe position of the liver fibrosis phantom. However, in a clinical setting, the ability to keep an organ motionless for the time taken to acquire several measurements in one loop is more limited, necessitating acquisition of several loops which might affect repeatability in vivo. Adjustment of the size of the measurement ROI by the observer might also potentially affect repeatability; however, this was standardized in our study.

We have demonstrated a nearly perfect interobserver agreement (Table 4) for all the four novel systems as well as for TE, and no significant difference in mean elasticity measurements between the observers (Table 3) for the novel systems. This is in line with previous reports from studies assessing the intra- and interobserver reliability in other shear wave systems that have been longer on the market, which have also concluded with a high repeatability for shear wave elastography *in vitro*, as well as *in vivo* in breast masses, healthy liver tissue in adults and children. [21–24]. In our study, the elasticity measurements of TE were to comparatively higher on phantom 4, and significantly present for both observers. A difference in experience with TE, between observer A and B, might be a possible factor. However, previous publications have stated that the slope of the curve for elastography measurements across increasingly hard phantoms or liver tissues may differ between elastography systems and platforms. Oudry et al. demonstrated that vibration-controlled transient elastography (TE) had a steeper slope, and elastography values increased more, between phantoms of increasing hardness compared to a shear wave technique, and discussed that this was in part due to the lower frequency and smaller size of the vibration source in TE leading to increased diffraction, an effect which is increased as materials get harder and which is known to induce overestimation of stiffness. [25]

Significant differences in shear wave velocity was demonstrated at different imaging depths (3–7 cm) in one study; and a significant difference in shear wave speed estimated among the systems was shown. However, due to the study design using different phantoms for different depths, the question of whether this systematic error was due to the imaging system or difference in the material properties of the phantom remained unanswered. [12] Another phantom study evaluated the repeatability of two elastography methods at depths 1–4 cm, and found that the depth related differences were small, but significant [26]. In our study, we used the same liver fibrosis phantoms and we subsequently performed all measurements at similar distance, approximately 3.5 cm inferior of the transducer surface.

The liver fibrosis phantoms were compatible with shear wave modalities, including TE and ARFI, however most of the phantom elasticity measurements obtained by the shear wave methods underestimated the elasticity values provided by the manufacturer of the phantom, especially for the softer phantoms. (Fig 4) This was equally observed by both observers, and may be caused by the in vitro material, although the phantom material density was 1.03g/cm^3^, which is comparable to live soft tissue. The liver fibrosis phantoms did not provide the same elastic properties as live soft tissue, the acoustic properties are similar and comparable to live soft tissue. It is previously shown that change in attenuation coefficient may affect the penetration results for ultrasound scanners and cause variations not related to the performance of the scanner. However, these changes would not have a significant effect. [27] It was beyond the scope of this paper to evaluate whether similar underestimation will occur when scanning live tissue, and this must be further investigated in vivo.

The present results in a homogeneous tissue-mimicking phantom material are promising for the successful clinical application of the novel shear wave methods from Hitachi, GE and Samsung; however, the study has some limitations inherent to its *in vitro* design. In a clinical setting, factors such as fasting status, narrow intercostal spaces, variable amounts of subcutaneous fat (affecting measurement depths) and variable patient cooperation (ability to maintain breath-hold) may affect results, which may also be influenced by variable levels of cholestasis, hepatic inflammation, ascites and other factors. The purely elastic phantoms do not accurately mimic the viscoelastic properties of human liver; however, no viscoelastic phantoms are currently commercially available to our knowledge. The phantoms we employed spanned relevant elasticities and had been subject to rigorous testing and quality control from the producer, and have been employed in similar studies of other systems. The *in vitro* design allowed us to standardize the default settings for phantom scanning, for all methods and both observers, and assess them without adjusting factors that may affect performance, such as depth of measurements. The homogenous and isotropic material of the phantoms is key for repeatability of measurements, but differ from the situation of scanning liver tissue *in vivo*.

## Conclusion

We have demonstrated similar and excellent repeatability and interobserver agreement for four novel SWE systems using liver tissue-mimicking phantoms. Further studies are needed to evaluate the performance of these methods in human liver scanning.

## Supporting information

S1 Study data This excel file includes the recorded raw data obtained by both observers, for each of the systems. (XLSX)
